# No evidence that relatedness or familiarity modulates male harm in *Drosophila melanogaster* flies from a wild population

**DOI:** 10.1002/ece3.8803

**Published:** 2022-04-11

**Authors:** Ana Marquez‐Rosado, Clara Garcia‐Co, Claudia Londoño‐Nieto, Pau Carazo

**Affiliations:** ^1^ 16781 Ethology Lab Cavanilles Institute of Biodiversity and Evolutionary Biology University of Valencia Valencia Spain

**Keywords:** kin discrimination, kin selection, sexual conflict

## Abstract

Sexual selection frequently promotes the evolution of aggressive behaviors that help males compete against their rivals, but which may harm females and hamper their fitness. Kin selection theory predicts that optimal male–male competition levels can be reduced when competitors are more genetically related to each other than to the population average, contributing to resolve this sexual conflict. Work in *Drosophila melanogaster* has spearheaded empirical tests of this idea, but studies so far have been conducted in laboratory‐adapted populations in homogeneous rearing environments that may hamper kin recognition, and used highly skewed sex ratios that may fail to reflect average natural conditions. Here, we performed a fully factorial design with the aim of exploring how rearing environment (i.e., familiarity) and relatedness affect male–male aggression, male harassment, and overall male harm levels in flies from a wild population of *Drosophila melanogaster*, under more natural conditions. Namely, we (a) manipulated relatedness and familiarity so that larvae reared apart were raised in different environments, as is common in the wild, and (b) studied the effects of relatedness and familiarity under average levels of male–male competition in the field. We show that, contrary to previous findings, groups of unrelated‐unfamiliar males were as likely to fight with each other and harass females than related‐familiar males and that overall levels of male harm to females were similar across treatments. Our results suggest that the role of kin selection in modulating sexual conflict is yet unclear in *Drosophila melanogaster,* and call for further studies that focus on natural populations and realistic socio‐sexual and ecological environments.

## Graphical abstract

Work in *Drosophila melanogaster* has spearheaded empirical test of the idea that kin selection may modulate sexual conflict. However, studies so far have focused on laboratory‐adapted populations in conditions that do not reflect average conditions in the wild. We replicated past experiments using a wild population and more natural conditions and did not find evidence that relatedness or familiarity modulate male harm to females.

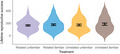

## 1 INTRODUCTION

Strong intrasexual competition in males frequently gives rise to male adaptations that harm females. Such “male harm” comes about through a staggering diversity of adaptations across many different taxa and can have crucial evolutionary implications for females and populations by modulating dispersal, driving intersexual coevolution, and decreasing population productivity (Arnqvist & Rowe, 2005; Candolin, 2019; Le Galliard et al., 2005; Pizzari et al., 2015). Currently, however, we are far from understanding the extraordinary variability in the existence and intensity of male harm traits across the tree of life.

Over the last decade, theoretical advances have suggested that kin selection might be an important factor to unravel the evolution and diversity of male harm (Faria et al., 2015, 2020; Pizzari et al., 2015; Rankin, 2011; Wild et al., 2011). The original models generally show that population structure can modulate the evolution of male harm such that, when competing against related males, harming a common resource can negatively impact a male's own inclusive fitness, and hence, kin selection may push males to decrease harm to females and ameliorate sexual conflict (Faria et al., 2015; Rankin, 2011; Wild et al., 2011). More recently, theoretical studies have revealed further nuance in the interplay between sexual selection and sexual conflict, with potentially wide‐ranging repercussions. For example, Faria et al. (2020) show that, while kin discrimination can inhibit harmful male behaviors to females at an individual level (i.e., when competing against related males), it may actually intensify sexual conflict at the population level because it makes males disproportionally more harmful to females when competing against unrelated males. Group size is also predicted to modulate kin selection effects on sexual conflict, such that male modulation of male harm in response to kin is expected to decrease with increasing group size (Pizzari et al., 2015). Finally, it has also been shown that sex‐specific relatedness patterns can lead to differences in how related a male is to his group mates with respect to his maternal‐origin vs. paternal‐origin genes, such that resulting intra‐genomic conflict may drive the evolution of genomic imprinting (Faria et al., 2017). Inclusive fitness ideas had been previously applied to some forms of pre‐copulatory male–male reproductive cooperation in a few social species (Díaz‐Muñoz et al., 2014; Krakauer, 2005). Yet, this new theory crucially means that kin selection could potentially play a much vaster role in modulating sexual conflict; from social to non‐social organisms, during pre‐ and post‐copulatory competition (essentially at all reproductive levels where male–male intrasexual selection may lead to male harm to females), and even at the intra‐genomic level.

Empirical studies have followed suit over the past few years, with reports congruent with the idea that relatedness modulates male harm accumulating in flies (*Drosophila melanogaster*; Carazo et al., 2014; Carazo et al., 2015; Hollis et al., 2015; Le Page et al., 2017), seed beetles (*Callosobruchus maculatus*; Lymbery & Simmons, 2017, 2020, but see Berg et al., 2019), bulb mites (*Rhizoglyphus robini*; Lukasiewicz et al., 2017), spider mites (*Tetranychus urticae*; Rodrigues et al., 2021), red junglefowl (*Gallus gallus*; Rosher et al., 2017; Tan et al., 2016), and least killifish (*Heterandria formosa* Ala‐Honkola et al., 2011). Such evidence includes proof of male plastic responses to relatedness reliant on kin discrimination (i.e., in flies, seed beetles, and red junglefowl (Carazo et al., 2014; Lymbery & Simmons, 2017, 2020; Le Page et al., 2017; Rosher et al., 2017; Tan et al., 2016), as well as nonplastic responses to experimental evolution under increased local relatedness (i.e., in mites; Lukasiewicz et al., 2017; Rodrigues et al., 2021). There is thus accumulating evidence that the evolutionary interplay between kin selection and sexual conflict is an emerging research area with the potential to advance our understanding of female harm, sexual cooperation, intra‐genomic conflict, and male and female co‐evolution at large.

In this context, *Drosophila melanogaster* has the potential to become a model system in the study of kin selection and sexual conflict. It is already a model system in sexual conflict studies, exhibiting intense sexual conflict that includes both precopulatory (i.e., male harassment; Teseo et al., 2016) and post‐copulatory (i.e., toxic ejaculates (Wigby & Chapman, 2004, 2005)) harm to females. It is also capable of kin recognition in both its larval (Khodaei & Long, 2019) and adult stages (García‐Roa et al., 2022; Lize et al., 2013; Tan et al., 2013). Moreover, previous evidence has shown evidence of cooperation among kin (Khodaei & Long, 2019, 2020), and it is the first species in which males were shown to decrease male harm when competing against related males (Carazo et al., 2014). Unfortunately, it is yet unclear whether kin selection is of any relevance to understand the evolution of sexual conflict in *Drosophila melanogaster*. First, because evidence that males of this species modulate male–male competition and harm to females in the presence of related males is equivocal, seemingly depending on the population of origin. While original reports that males modulate male harm in response to local relatedness have been successfully replicated several times in the laboratory‐adapted Dahomey population of sub‐Saharan origin (collected in 1970 in Benin, Africa (Carazo et al., 2014, 2015; Le Page et al., 2017)), studies conducted with the IV population of cosmopolitan origin (collected in 1975, in Massachusetts, USA) have failed to mirror these findings (Chippindale et al., 2015; Martin & Long, 2015), but see (Hollis et al., 2015).

Second, because *Drosophila* studies have so far focused on laboratory‐adapted populations and socio‐sexual contexts that may not reflect natural conditions well. On the one hand, there is conclusive evidence suggesting that both environmental and genetic cues are important for kin recognition in *D*. *melanogaster* (Lize et al., 2013), including recent studies on “sexual altruism” (Hollis et al., 2015; Le Page et al., 2017). However, to date, studies have examined the impact of environmental cues by comparing the behavior of flies reared in the same vs. different vials (i.e., familiar vs. unfamiliar), but using the exact same rearing environment across treatments (i.e., same recipe and usually even batch of food). These conditions differ markedly from what is expected to happen in nature, where flies from different clutches (whether from the same or different mothers) will normally develop in different rearing environments due to variation in available fruit types, fruit size, fruit ripeness, density of larvae etc., as well as to inter‐individual variation in oviposition preferences (Dweck et al., 2013; Mansourian et al., 2018). For example, Dweck et al. (2013) showed that fruit flies exhibit a clear preference for citrus fruits, but that despite this approximately 40%–60% flies still lay their eggs on alternative fruit. Furthermore, such variability was detected even though fruits were carefully controlled for ripeness and external damage (inducing differences in yeast content), which is unlikely to be the case in nature (Dweck et al., 2013). In addition, gut microbiota has been shown to play an important role in kin recognition in *D*. *melanogaster* (García‐Roa et al., 2022; Heys et al., 2018; Lize et al., 2013). Gut microbiota is transferred into the medium by females during oviposition, and is heavily influenced by environmental factors, including the food regime flies are kept in (Broderick & Lemaitre, 2012; García‐Roa et al., 2022). Consequently, the gut microbiome of laboratory flies kept under the same food is bound to exhibit considerably less inter‐individual variation than in nature. In short, by inadvertently controlling for environmental cues that would be present in nature (and that have been shown to influence mate choice in *D*. *melanogaster* (Sharon et al., 2011)), past studies may have curtailed the potential and/or efficacy of kin/familiarity recognition mechanisms in this species.

On the other hand, studies have so far focused on studying how relatedness and familiarity modulate male harm under particularly high sexual conflict, where three males constantly compete for access to a single female. While this operational sex ratio is certainly not uncommon in the wild, it seems to represent the high end of the male–male competition spectrum (Dukas, 2020). Thus, new studies with more natural populations and under more realistic conditions seem vital to properly gauge how relevant kin selection may be to sexual conflict in *D*. *melanogaster*. To this end, we set up an experiment using flies from a recently sampled wild population of *D*. *melanogaster*. We factorially manipulated relatedness (i.e., unrelated vs. full‐sibs) and familiarity (i.e., reared together vs. apart) of two rival males competing for access to an unrelated female, thus reflecting a 2:1 operative sex ratio that is close to average ratios observed in nature (Dukas, 2020). In addition, we manipulated the larval rearing environment so that unfamiliar flies were always raised in different food, simulating natural conditions and maximizing the potential for kin/familiarity recognition to occur. If kin selection plays an important role driving male harm, we would expect relaxed male–male competition, male harassment of females, and higher female fitness in related‐familiar treatments (i.e., full‐sibs reared together) vs. unrelated‐unfamiliar flies (i.e., unrelated and reared apart).

## 2 MATERIALS AND METHODS

### 2.1 Study population

We used a natural population of *Drosophila melanogaster*, called “Vegalibre (VG).” This population was collected in October 2018, in the exterior of three wineries in Requena (Valencia, Spain): “Hispano‐Suizas” (39.466128, −1.149642), “Pago de Tharsys” (39.49834, −1.122781), and “Dominio de la Vega” (39.515079, −1.143757) and has since been kept outbred at a population size of >2000 flies, to which we add flies from between 50 and 100 isofemale lines that are annually re‐sampled at the same original locations. In the laboratory, VG population flies are maintained under a temperature fluctuation schedule that mimics natural conditions in the field (24°C mean temperature fluctuating ±4°C during each cycle of 24 h), 60% humidity, and a 12:12 dark–light cycle. The standard medium used to feed the population consists of 6.76 g/L of agar, 72 g/L of maize, 14.64 g/L of yeast, 8.64 g/L of soya, 72 g/L of malt, 20 g/L of molasses, 33 ml of Nipagin mix (2.96 g/L of Nipagin, 28.12 ml of ethanol and 1.48 ml of water) and 5.6 ml of acid mix (95% propionic and 5% phosphoric).

### 2.2 Experimental design

Our overarching aim was to evaluate whether relatedness (i.e., full‐sibs vs. unrelated) and familiarity (i.e., flies reared together vs. apart as larvae) had an impact on male‐male competition and male harassment to females and particularly on female fitness. In order to do so, we conducted a series of behavioral and fitness assays in which we exposed virgin females to pairs of virgin males that were: (a) full‐sibs and had been reared together (“related‐familiar”), (b) full‐sibs and raised apart (“related‐unfamiliar”), (c) unrelated and had been reared together (“unrelated‐familiar”), and (d) unrelated and raised apart (“unrelated‐unfamiliar”). To mimic natural spatial heterogeneity in the rearing conditions of different clutches, we also manipulated larval rearing environment so that unfamiliar flies were always from different rearing media: mashed banana or mashed apple (a preliminary pilot study confirmed that eggs developed similarly well under these two conditions; see Appendix S1).

We first generated parental flies (later used to produce families, see below) by collecting eggs from our VG stock population on grape‐agar filled Petri dishes smudged with live yeast paste. We placed eggs at a standardized density of 15 µl per 75 ml bottle containing ~45 ml of standard medium following the protocol described by (Clancy and Kennington, 2001). We then left flies to incubate at 24°C until emergence, at which time we collected them as virgins (i.e., <8 h post‐eclosion) using ice anesthesia, sexed them, and kept in vials with standard medium until their use (~6 days old). We then haphazardly paired a single virgin male and female for 24 h in individual vials containing cotton moistened with water, so as to provide moisture while preventing oviposition. Twenty‐four hours later, we discarded males and transferred females to vials with 27 ml of standard medium and live yeast (to induce female oviposition). We subsequently transferred females to fresh vials every 24 h for a total of three days, and immediately following each flip, we haphazardly transferred the eggs to one of two experimental rearing media (banana or apple based) according to the following protocol.

We obtained a total of 304 families, each with between 30 and 40 eggs (replicates for which parental females failed to produce a minimum of 30 eggs were discarded), that we divided into two groups, one with 124 families used to generate the stock of flies that we used for the “related” treatments (related‐familiar and related‐unfamiliar), the other with the 140 families, used to generate the stock of flies to create the “unrelated” treatments (unrelated‐familiar and unrelated‐unfamiliar), and 40 vials as backup. In the first group, eggs from each family were collected and divided equally into two vials differing in rearing environment. Thus, each family contributed 15–20 eggs to each of two vials (one with mashed banana and one with mashed apple; Figure S1). We then incubated all vials at 24°C and collected virgin males that were immediately housed in haphazardly allocated treatment vials with standard medium for 2–5 days (ensuring males were mature and socially familiar with their rivals as adults before the start of assays). Thus, related‐familiar treatments contained two virgin full‐sib males reared together in the same vial, while related‐unfamiliar treatments contained two virgin full‐sib males reared in different vials with different rearing media.

In the second group, we used flies from different families to generate mixed family vials for unrelated treatments by allocating one egg of each of 15 different families to 20 “banana” vials and 20 “apple” vials (see Figure S2), so that each pair of vials contained 20 eggs from unrelated family vials (following Le Page et al., 2017). We then incubated all vials at 24°C and collected virgin males as described above. Hence, unrelated‐familiar treatments contained two males from different families reared together on a mixed family vial (obviously, with the same rearing medium), and unrelated‐unfamiliar treatments contained two males from different families reared in different vials/rearing media (Figure S2). We alternated the food used to raise familiar males in. Our final sample sizes for each treatment were 75 vials for the related‐familiar treatment, 72 vials for the related‐unfamiliar treatment, 54 vials for the unrelated‐familiar treatments, and 64 vials for the unrelated‐unfamiliar treatments.

To produce virgin experimental females, we collected eggs from the VG population stock (following the method described above) in separate bottles to those used to collect experimental males. We then collected them as virgins and maintained them in groups of 15 in vials for 3–5 days at 24°C, until the beginning of assays. All females were unrelated to experimental males.

### 2.3 Behavioral and fitness assays

Assays started by introducing pairs of experimental males with one experimental female into a fresh vial. We then followed triplets for three weeks of interaction, then discarded males, and followed female in individual vials until they died. For the first three weeks of interaction, we used mild CO_2_ anesthesia to flip triplets to fresh vials every 3–4 days (i.e., twice a week), then incubated vials containing eggs for 16 days at 24°C to ensure all adults emerged, and froze those vials to eventually count offspring. After we discarded males, we continued to flip females and to incubate their offspring until they died. Ultimately, this allowed us to estimate lifetime reproductive success and survival of experimental females.

In addition, during the first three days of each week of the interaction period, we conducted behavioral observations for three hours daily after lights on at 9 a.m. Vials were scanned (3s per vial) once every 15 min, for a total of 12 scans per vial per day of observation. We scored all courtships, male–male aggressions, female rejection behaviors and matings (see Table S1 for an ethogram). Specifically, we measured (a) male harassment/courtship rate/intensity (i.e., the average number of males courting a female per scan; Bastock & Manning, 1955), (b) male–male aggression rates (i.e., the average number of male‐male aggression events observed per scan; Chen et al., 2002), (c) the total number of mating events, and (d) rejection rate (i.e., the total number of rejection events per scans; Bastock & Manning, 1955; Connolly & Cook, 1973). Behavioral observations were conducted by two observers (CGC and PC) after previously conducting adequate intra‐ and inter‐observer reliability trials (Martin & Bateson, 1993). During all the experiments, we checked vials daily for mortality until females died. In the event of a male dying during the first three weeks of interaction, we right‐censored vials (for survival analysis) and excluded them from calculations of female lifetime reproductive fitness.

### 2.4 Statistical analyses

To evaluate relatedness and familiarity effects on female lifetime reproductive success (hereafter LRS), we used generalized linear models (GLMs) with relatedness, familiarity, and their interaction as fixed factors, using type III ANOVA to calculate *p*‐values. When necessary, data were winsorized to avoid the potential undue influence of extreme values. However, note that analyzing nontransformed data produces qualitatively identical results. After model fitting, we examined diagnostic plots to evaluate normality and heteroscedasticity assumptions. To analyze the effect of relatedness, familiarity and their interaction on survival (i.e., female lifespan), we fitted a Cox proportional hazards model. Behavioral variables were first transformed into rates per minute (in the case of courtship, aggression, and rejection rates) and matings per observation day (3 h). We then run GLMs with behavioral rates as response variables and relatedness, familiarity, and their interaction as fixed factors. However, graphic analysis of diagnostic plots confirmed heteroscedasticity and normality violations that we could not circumvent using non‐Gaussian distributions (e.g., beta‐binomial). We thus proceeded to analyze data using nonparametric Kruskal–Wallis tests. Like with fitness variables, when necessary, we winsorized to minimize the influence of existing outliers, but again note that data with and without winsorizing were qualitatively identical. We also note that results obtained using nonparametric analyses were always qualitatively identical to those obtained in the initial GLMs.

## 3 RESULTS

**FIGURE 1:**
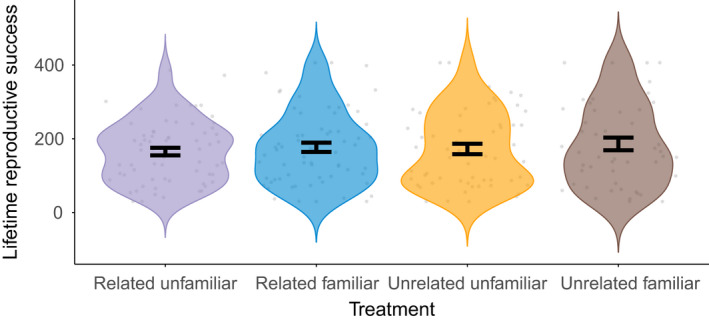
Lifetime reproductive success of females across treatments. Points show the total number of offspring from each female during the experimental period and the bars (in black) show the mean ± SEM

**FIGURE 2:**
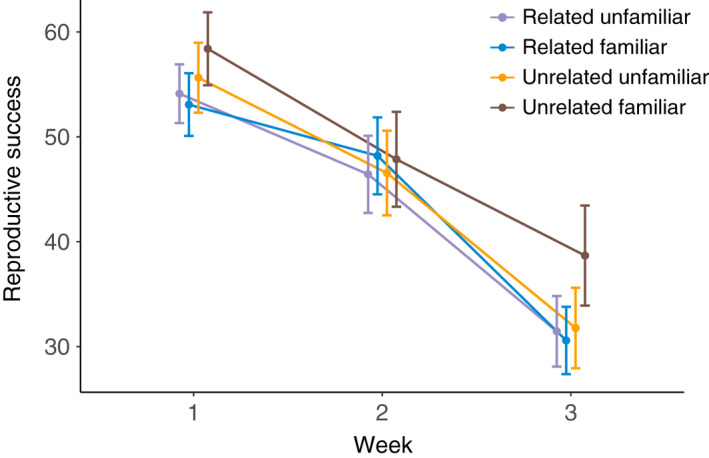
Female reproductive success per week (for the first three weeks) across treatments (mean ± SEM)

**FIGURE 3:**
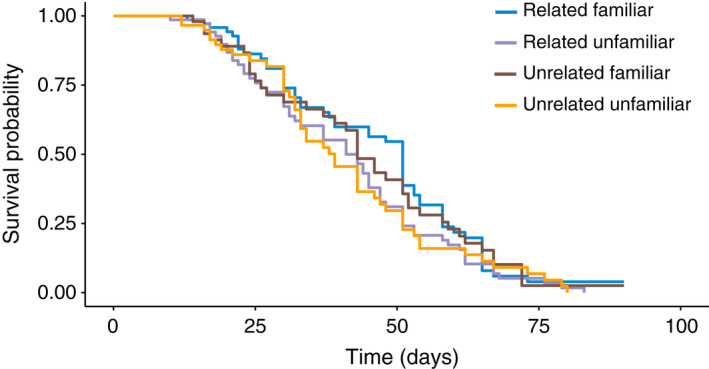
Female survivorship curve across treatments

For female lifetime reproductive success, we did not find evidence of a significant interaction between relatedness and familiarity (*F*_1,239_ = 0.006, *p* = .935, coefficient = −0.52 ± 12.5; mean ± 95% CI) or of a main effect of relatedness (*F*_1,239_ = 0.209, *p* = .648, coefficient = 2.90 ± 12.49; mean ± 95% CI) or of familiarity (*F*_1,239_ = 0.739, *p* = .391, coefficient = −5.45 ± 12.48; mean ± 95% CI) (Figures 1 and 2). Similarly, we did not find a significant interaction in female survival across treatments (Wald test for overall model, *z* = 2.95, df = 3, *p* = .4; interaction, *z* = −0.424, *p* = .672; Figure 3, coefficient = 0.97 with a 95% CI of 0.84–1.12), nor evidence of a significant effect of relatedness (*z* = −0.202, *p* = .840, coefficient = 0.98 with a 95% CI of 0.86–1.13), or familiarity (*z* = −1.650, *p* = .099, coefficient = 0.89 with a 95% CI of 0.77–1.02).

**FIGURE 4:**
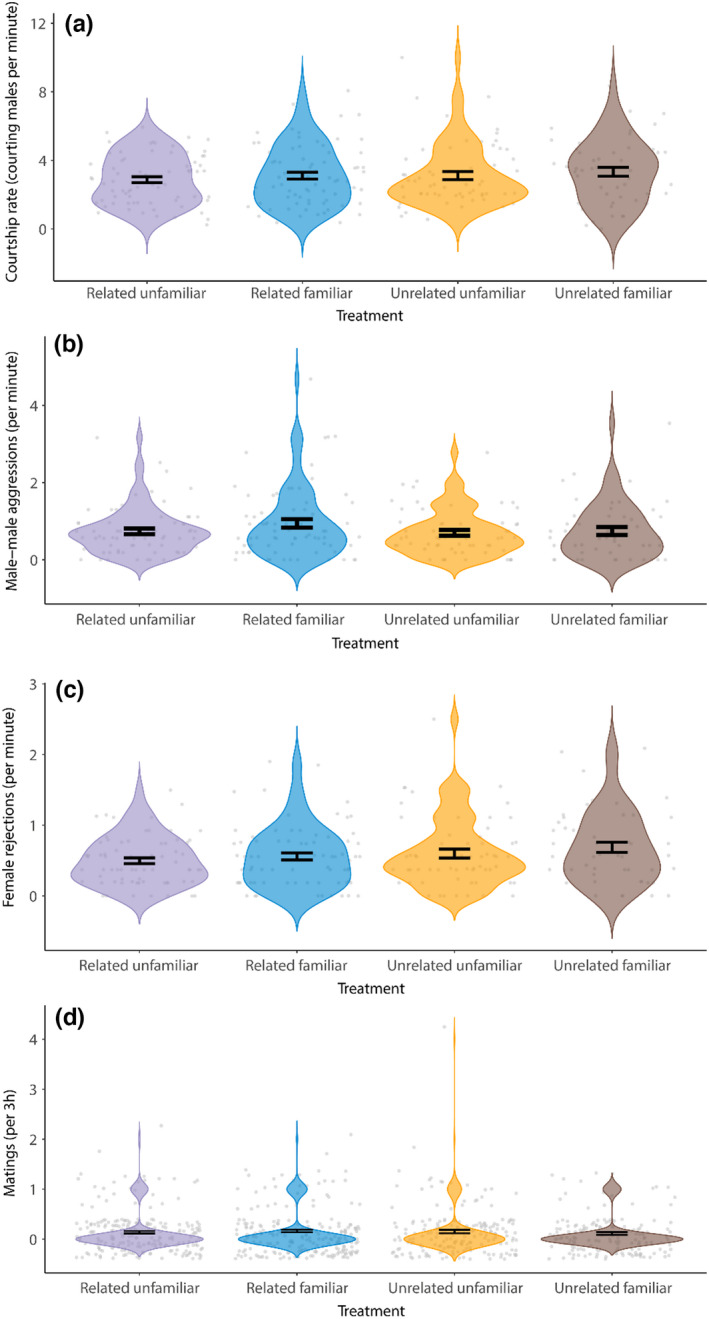
Behavioral rates across treatments (mean ± SEM): (a) male harassment of females (i.e., courtship rates), (b) male–male competition level (i.e., male–male aggression rates), (c) female resistance to male harassment (i.e., rejection rate), and (d) overall mating rate

As in the case of fitness variables, we found no evidence of significant differences in behavioral variables across treatments (Figure 4), in either the intensity of male‐male aggression (df = 3, X^2^ = 2,02, *p* = .568), courtship activity (df = 3, X^2^ = 1,577, *p* = .665), mating (df = 3, X^2^ = 2,106, *p* = .551), or on female rejection rates (df = 3, X^2^ = 3,4473, *p* = .328).

## 4 DISCUSSION

We did not find any evidence that, under our experimental conditions, within‐group relatedness and/or familiarity modulated male harm to females. To date, several experiments have looked at whether relatedness/familiarity modulate male harm to females in *D*. *melanogaster*, with mixed results. The first demonstration of this phenomena reported that *D*. *melanogaster* male flies from the Dahomey population were both less aggressive and less harmful to females when competing in groups of related males, with a significant boost in terms of estimated population productivity (Carazo et al., 2014). A follow‐up study in the same population replicated the finding that males harassed females less and were generally less aggressive to each other when in the presence of related males and that this had positive fitness transgenerational effects on daughters (Carazo et al., 2015). Hollis et al. (2015) replicated these results in flies from a different strain (population IV), critically showing that familiarity was necessary for such “sexual altruism” to occur (i.e., related flies also had to be raised in the same vial). Le Page et al. (2017) followed this up in Dahomey, in a fully factorial experiment combining relatedness and familiarity, to show that both were necessary to elicit modulation of male harm. The latter results fit well with the frequent finding that both environmental (i.e., shared environment), and genetic cues are normally implicated in kin recognition (Davies et al., 2012). In contrast to the above studies, two additional experiments by Chippindale et al. (2015) and Martin and Long (2015), both using population IV flies kept under a 10d, non‐overlapping generation cycle in the laboratory for thousands of generations, failed to replicate these results. Our result adds to these latter studies, raising the question as to how to properly interpret available results and whether kin selection is at all important to understand male harm in *D*. *melanogaster*.

The first possibility is that reported effects that relatedness/familiarity modulate harm to females in *D*. *melanogaster* have not evolved due to its inclusive fitness benefits. Pizzari et al. (2015) suggested the possibility that males may perceive related/familiar males as weaker perceptions of themselves. Thus, reduction in male–male competition and harm to females in groups of related males may actually reflect a generalized Coolidge effect, if males conflate females mated with related/familiar males as mated with themselves. Or it may simply reflect male modulation of investment in reproduction in response to a lower perceived level of sperm competition risk/intensity, if males conflate odors that are more similar as reflecting a lower number of competing males (Pizzari et al., 2015). This latter possibility has also been proposed for seed beetles (Lymbery & Simmons, 2020), but is very difficult to tease apart from the kin selection hypothesis because many insects rely on odors to assess sperm competition levels (Carazo et al., 2012; Shifferman, 2012), and thus, it seems that relatedness/familiarity and competition level may be inextricable. In addition, the possibility that responses to relatedness/familiarity may not have evolved in response to kin selection does not mean they cannot be exapted for their inclusive fitness benefits, whenever there is adequate population structure (Pizzari et al., 2015). It is also worth noting that, irrespective of the functional origin of these plastic responses, we would still have to explain the discordance among *D*. *melanogaster* studies.

A second possible explanation could have to do with population‐of‐origin effects. There is considerable evidence of marked molecular and behavioral differentiation, and even incipient reproductive isolation, between African and cosmopolitan (i.e., flies expanded to the rest of the world from its ancestral African range via human transport) populations of *D*. *melanogaster* (Cohet & David, 1980; Pool et al., 2012; Wu et al., 1995). Thus, while Carazo et al. (2015) and Le Page et al. (2017) used the Dahomey population, which was collected in Dahomey (Benin) in 1970 and has been maintained since then in the laboratory (Partridge & Farquhar, 1983), Chippindale et al. (2015) and Martin and Long (2015) used the cosmopolitan IV population, which was captured in 1975 in Massachusetts (USA) (Charlesworth & Charlesworth, 1985) and has also been maintained in the lab since then. Our experiment was also conducted with flies from a cosmopolitan wild population recently sampled in Valencia (Spain), so it could be that the disparity in the outcome of these two groups of studies reflect variation between the ancestral and cosmopolitan lineages. However, this hypothesis does not fit with results from Hollis et al. (2015), which found clear effects of relatedness/familiarity on male harm to females using population IV. The only notable difference between these population IV strains is that both Chippindale et al. (2015) and Martin and Long (2015) used populations kept under 14 days and 28 days (respectively) discrete generation cycles (i.e., under short, non‐overlapping generations for thousands of generations), a regime that may not allow for the accumulation of mid‐to‐late‐life female harm effects observed that seem to drive relatedness effects (Carazo et al., 2014, 2015; Hollis et al., 2015; Le Page et al., 2017).

In addition to the above, our findings may also be partly explained by the fact that we staged male–female interactions using a 2:1 sex ratio that is considerably less extreme than the 3:1 ratio used thus far. Our experiment was conservatively designed to gauge kin effects in the lower range of variation in male–male competition that seems typical of this species in nature (Dukas, 2020). Specifically, Dukas (2020) recently reported that, in a natural cosmopolitan population observed in the wild, the average sex ratio of flies at fruits is close to 2:1 across early morning observations, which is the period of peak sexual activity during the day. We also limited the interaction phase of our experiment to three weeks, so that females were only exposed to males during the most active reproductive part of their lives. This is in contrast with previous studies, which allowed male harassment to proceed throughout the entire female lifespan. In addition, and due to the practical limitations involved in the fully factorial design of our experiment (see also Le Page et al., 2017), we could not replace competitor males to prevent the effects of co‐aging, as in the original experiments (Carazo et al., 2014, 2015). This means that, if males aged quicker in vials where male‐male competition (and harm) were higher, relatedness/familiarity effects may have waned through time due to differential male aging across treatments, although note this same limitation did not prevent Le Page et al. (2017) from finding relatedness/familiarity effects. All in all, our design is thus indicative of what may happen in low‐moderate conflict scenarios in nature, and it is entirely possible that relatedness/familiarity only modulate harm to females significantly in situations of high sexual conflict.

As a more general corollary, our results further underscore the importance of expanding laboratory‐based studies to natural conditions. Too few studies aim to replicate ground‐breaking findings, frequently necessarily conducted under artificial settings, in more biologically relevant scenarios that better reflect natural conditions. Here, we aimed to study the potential role of relatedness in modulating male harm under conditions that more accurately represent average conditions in nature (compared to available studies), in our case by imposing lower sex ratios and adding heterogeneity in the rearing environment. Our results join those of other recent studies (Yun et al., 2017, 2018) in highlighting how such replicates can be critical for a comprehensive understanding of behavioral phenomena, including sexual selection processes that have been solely studied in artificial and/or a subset of ecologically relevant conditions.

## 5 CONCLUSIONS

Strong intrasexual competition in males frequently gives rise to male adaptations that harm females. Such “female harm” comes about through a staggering diversity of adaptations across many different taxa and can have crucial evolutionary implications for females and populations by modulating dispersal, driving intersexual coevolution, and decreasing population productivity. Currently, however, we are far from understanding the extraordinary variability in the existence and intensity of male harm across the tree of life. Recent theoretical advances have suggested that kin selection might be an important factor to fill this gap in knowledge, with *D*. *melanogaster* emerging as a model system in this area of research thanks to a handful of pioneering studies. However, available evidence has so far been based on work conducted exclusively in laboratory‐adapted populations, under extreme levels of sexual conflict, and not taking into account variation in larval rearing environment across different clutches of eggs, which may be important to allow for kin discrimination in this species. Here, we replicated the key experimental design used so far while taking the aforementioned factors into account. We found little evidence that, under this more natural scenario, relatedness or familiarity modulated male harm to females in flies from a recently sampled wild *D*. *melanogaster* population. Our results caution against hasty interpretations about the relevance of kin selection to understand male harm evolution in *Drosophila* and suggest future studies should focus more on natural populations and a wider range of natural socio‐sexual contexts (e.g., varying levels of male‐male competition, spatial structure, and variation in density).

## CONFLICT OF INTEREST

We declare no conflicts of interest.

## AUTHOR CONTRIBUTIONS

**Ana Marquez‐Rosado:** Data curation (equal); Formal analysis (equal); Investigation (equal); Methodology (equal); Validation (equal); Visualization (equal); Writing – review & editing (equal). **Clara Garcia‐Co:** Data curation (equal); Formal analysis (equal); Investigation (equal); Methodology (equal); Validation (equal); Visualization (equal); Writing – review & editing (equal). **Claudia Londoño‐Nieto:** Investigation (supporting); Methodology (supporting); Supervision (supporting); Writing – review & editing (supporting). **Pau Carazo:** Conceptualization (lead); Data curation (supporting); Formal analysis (equal); Funding acquisition (lead); Investigation (supporting); Methodology (equal); Resources (lead); Supervision (lead); Validation (equal); Visualization (equal); Writing – original draft (lead).

## Supporting information

Appendix S1

## Data Availability

All data will be made available in a public repository and/or as supplementary material as soon as the paper is accepted for publication. Data and code will also be available for reviewers upon demand.

## References

[ece38803-bib-0001] Ala‐Honkola, O. , Friman, E. , & Lindstrom, K. (2011). Costs and benefits of polyandry in a placental poeciliid fish *Heterandria formosa* are in accordance with the parent‐offspring conflict theory of placentation. Journal of Evolutionary Biology, 24(12), 2600–2610. 10.1111/j.1420-9101.2011.02383.x 21902749

[ece38803-bib-0002] Arnqvist, G. , & Rowe, C. (2005). Sexual conflict. Princeton University Press.

[ece38803-bib-0047] Bastock, M. , & Manning, A. (1955). The courtship of *Drosophila Melanogaster* . Behaviour, 8(1), 85–110.

[ece38803-bib-0003] Berg, E. C. , Lind, M. I. , Monahan, S. , Bricout, S. , & Maklakov, A. A. (2019). Kin but less than kind: Within‐group male relatedness does not increase female fitness in seed beetles. Proceedings of the Royal Society B: Biological Sciences, 286(1910), 20191664. 10.1098/rspb.2019.1664 PMC6742989 31506055

[ece38803-bib-0004] Broderick, N. A. , & Lemaitre, B. (2012). Gut‐associated microbes of *Drosophila melanogaster* . Gut Microbes, 3(4), 307–321. 10.4161/gmic.19896 22572876 PMC3463489

[ece38803-bib-0005] Candolin, U. (2019). Sexual selection and sexual conflict. In B. Fath (Ed.), Encyclopedia of ecology (2nd ed., pp. 310–318). Elsevier.

[ece38803-bib-0006] Carazo, P. , Fernández‐Perea, R. , & Font, E. (2012). Quantity estimation based on numerical cues in the mealworm beetle (*Tenebrio molitor*). Frontiers in Psychology, 3, 502. 10.3389/fpsyg.2012.00502 23372554 PMC3555739

[ece38803-bib-0007] Carazo, P. , Perry, J. C. , Johnson, F. , Pizzari, T. , & Wigby, S. (2015). Related male *Drosophila melanogaster* reared together as larvae fight less and sire longer lived daughters. Ecology and Evolution, 5(14), 2787–2797. 10.1002/ece3.1549 26306167 PMC4541986

[ece38803-bib-0008] Carazo, P. , Tan, C. K. W. , Allen, F. , Wigby, S. , & Pizzari, T. (2014). Within‐group male relatedness reduces harm to females in Drosophila. Nature, 505(7485), 672–675. 10.1038/nature12949 24463521 PMC5768239

[ece38803-bib-0048] Charlesworth, B. , & Charlesworth, D. (1985). Genetic variation in recombination in *Drosophila*. I. Responses to selection and preliminary genetic analysis. Heredity, 54(1), 71–83.

[ece38803-bib-0049] Chen, S. , Lee, A. Y. , Bowens, N. M. , Huber, R. , & Kravitz, E. A. (2002). Fighting fruit flies, a model system for the study of aggression. PNAS, 99, 5664–5668. 11960020 10.1073/pnas.082102599 PMC122828

[ece38803-bib-0009] Chippindale, A. K. , Berggren, M. , Alpern, J. H. , & Montgomerie, R. (2015). Does kin selection moderate sexual conflict in Drosophila? Proceedings of the Royal Society B: Biological Sciences, 282(1813), 1417. 10.1098/rspb.2015.1417 PMC4632634 26269501

[ece38803-bib-0050] Clancy, D. J. , & Kennington, J. (2001). A simple method to achieve consistent larval density in bottle cultures. Drosophila Information Service, 84, 168–169.

[ece38803-bib-0010] Cohet, Y. , & David, J. R. (1980). Geographic divergence and sexual behaviour: Comparison of mating systems in French and afrotropical populations of *Drosophila melanogaster* . Genetica, 54(2), 161–165. 10.1007/BF00055986

[ece38803-bib-0051] Connolly, K. , & Cook, R. (1973). Rejection responses by female *Drosophila melanogaster*, Their ontogeny, causality and effects upon the behaviour of the courting male. Behaviour, 44(1), 142–165.

[ece38803-bib-0011] Davies, N. B. , Krebs, J. R. , & West, S. A. (2012). An introduction to behavioural ecology (4th ed.). Wiley‐Blackwell.

[ece38803-bib-0012] Díaz‐Muñoz, S. L. , DuVal, E. H. , Krakauer, A. H. , & Lacey, E. A. (2014). Cooperating to compete: Altruism, sexual selection and causes of male reproductive cooperation. Animal Behaviour, 88, 67–78. 10.1016/j.anbehav.2013.11.008

[ece38803-bib-0013] Dukas, R. (2020). Natural history of social and sexual behavior in fruit flies. Scientific Reports, 10(1), 21932. 10.1038/s41598-020-79075-7 33318613 PMC7736333

[ece38803-bib-0014] Dweck, H. K. M. , Ebrahim, S. A. M. , Kromann, S. , Bown, D. , Hillbur, Y. , Sachse, S. , Hansson, B. S. , & Stensmyr, M. C. (2013). Olfactory preference for egg laying on citrus substrates in Drosophila. Current Biology, 23(24), 2472–2480. 10.1016/j.cub.2013.10.047 24316206

[ece38803-bib-0015] Faria, G. S. , Gardner, A. , & Carazo, P. (2020). Kin discrimination and demography modulate patterns of sexual conflict. Nature Ecology & Evolution, 4(8), 1141–1148. 10.1038/s41559-020-1214-6 32451427 PMC7610387

[ece38803-bib-0016] Faria, G. S. , Varela, S. A. , & Gardner, A. (2015). Sex‐biased dispersal, kin selection and the evolution of sexual conflict. Journal of Evolutionary Biology, 28(10), 1901–1910. 10.1111/jeb.12697 26190034

[ece38803-bib-0017] Faria, G. S. , Varela, S. A. M. , & Gardner, A. (2017). Sexual selection modulates genetic conflicts and patterns of genomic imprinting. Evolution, 71, 526–540. 10.1111/evo.13153 27991659 PMC5347858

[ece38803-bib-0018] García‐Roa, R. , Domínguez‐Santos, R. , Pérez‐Brocal, V. , Moya, A. , Latorre, A. , & Carazo, P. (2022). Kin recognition in *Drosophila*: Rearing environment and relatedness can modulate gut microbiota and cuticular hydrocarbon odour profiles. Oikos. 10.1111/oik.08755

[ece38803-bib-0019] Heys, C. , Lizé, A. , Colinet, H. , Price, T. A. R. , Prescott, M. , Ingleby, F. , & Lewis, Z. (2018). Evidence that the microbiota counteracts male outbreeding strategy by inhibiting sexual signaling in females. Frontiers in Ecology and Evolution, 6, 29. 10.3389/fevo.2018.00029

[ece38803-bib-0020] Hollis, B. , Kawecki, T. J. , & Keller, L. (2015). No evidence that within‐group male relatedness reduces harm to females in Drosophila. Ecology and Evolution, 5(4), 979–983. 10.1002/ece3.1417 25750723 PMC4338979

[ece38803-bib-0021] Khodaei, L. , & Long, T. A. F. (2019). Kin recognition and co‐operative foraging in *Drosophila melanogaster* larvae. Journal of Evolutionary Biology, 32(12), 1352–1361. 10.1111/jeb.13531 31454451

[ece38803-bib-0022] Khodaei, L. , & Long, T. A. F. (2020). Kin recognition and egg cannibalism by *Drosophila melanogaster* larvae. Journal of Insect Behavior, 33(1), 20–29. 10.1007/s10905-020-09742-0

[ece38803-bib-0023] Krakauer, A. H. (2005). Kin selection and cooperative courtship in wild turkeys. Nature, 434(7029), 69–72. 10.1038/nature03325 15744300

[ece38803-bib-0024] Le Galliard, J. F. , Fitze, P. S. , Ferriäre, R. , & Clobert, J. (2005). Sex ratio bias, male aggression, and population collapse in lizards. Proceedings of the National Academy of Sciences of the United States of America, 102(50), 18231–18236. 10.1073/pnas.0505172102 16322105 PMC1312374

[ece38803-bib-0025] Le Page, S. , Sepil, I. , Flintham, E. , Pizzari, T. , Carazo, P. , & Wigby, S. (2017). Male relatedness and familiarity are required to modulate male‐induced harm to females in Drosophila. Proceedings of the Royal Society B: Biological Sciences, 284(1860), 20170441. 10.1098/rspb.2017.0441 PMC5563793 28794215

[ece38803-bib-0026] Lize, A. , McKay, R. , & Lewis, Z. (2013). Kin recognition in Drosophila: The importance of ecology and gut microbiota. ISME Journal, 8(2), 469–477. 10.1038/ismej.2013.157 PMC3906818 24030598

[ece38803-bib-0027] Lukasiewicz, A. , Szubert‐Kruszynska, A. , & Radwan, J. (2017). Kin selection promotes female productivity and cooperation between the sexes. Science Advances, 3, 31602262. 10.1126/sciadv.1602262 PMC5351977 28345048

[ece38803-bib-0028] Lymbery, S. J. , & Simmons, L. W. (2017). Males harm females less when competing with familiar relatives. Proceedings of the Royal Society B: Biological Sciences, 284(1867), 20171984. 10.1098/rspb.2017.1984 PMC5719177 29142115

[ece38803-bib-0029] Lymbery, S. J. , & Simmons, L. W. (2020). Gustatory cues to kinship among males moderate the productivity of females. Behavioral Ecology, 31(1), 81–89. 10.1093/beheco/arz158

[ece38803-bib-0030] Mansourian, S. , Enjin, A. , Jirle, E. V. , Ramesh, V. , Rehermann, G. , Becher, P. G. , Pool, J. E. , & Stensmyr, M. C. (2018). Wild African *Drosophila melanogaster* are seasonal specialists on Marula fruit. Current Biology, 28(24), 3960–3968.e3. 10.1016/j.cub.2018.10.033 30528579 PMC7065024

[ece38803-bib-0031] Martin, E. S. , & Long, T. A. F. (2015). Are flies kind to kin? The role of intra‐ and inter‐sexual relatedness in mediating reproductive conflict. Proceedings of the Royal Society B: Biological Sciences, 282(1821), 20151991. 10.1098/rspb.2015.1991 PMC4707750 26674954

[ece38803-bib-0032] Martin, P. , & Bateson, P. (1993). Measuring behaviour (vol. 2). Cambridge University Press.

[ece38803-bib-0052] Partridge, L. , & Farquhar, M. (1983). Lifetime mating success of male fruitflies *(Drosophila melanogaster)* is related to their size. Animal Behaviour, 31(3), 871–877.

[ece38803-bib-0033] Pizzari, T. , Biernaskie, J. M. , & Carazo, P. (2015). Inclusive fitness and sexual conflict: How population structure might modulate the battle of the sexes. BioEssays, 37, 155–166. 25389109 10.1002/bies.201400130

[ece38803-bib-0034] Pool, J. E. , Corbett‐Detig, R. B. , Sugino, R. P. , Stevens, K. A. , Cardeno, C. M. , Crepeau, M. W. , Duchen, P. , Emerson, J. J. , Saelao, P. , Begun, D. J. , & Langley, C. H. (2012). Population genomics of sub‐saharan *Drosophila melanogaster*: African diversity and non‐African admixture. PLoS Genetics, 8(12), e1003080. 10.1371/journal.pgen.1003080 23284287 PMC3527209

[ece38803-bib-0035] Rankin, D. J. (2011). Kin selection and the evolution of sexual conflict. Journal of Evolutionary Biology, 24(1), 71–81. 10.1111/j.1420-9101.2010.02143.x 21054623

[ece38803-bib-0036] Rodrigues, L. , Torralba Sáez, M. , Alpedrinha, J. , Lefèvre, S. , Brengues, M. , Magalhães, S. , & Duncan, A. B. (2021). Consequences of population structure for sex allocation and sexual conflict. Journal of Evolutionary Biology, 34(3), 525–536. 10.1111/jeb.13755 33314358

[ece38803-bib-0037] Rosher, C. , Favati, A. , Dean, R. , & Lovlie, H. (2017). Relatedness and age reduce aggressive male interactions over mating in male domestic fowl. Behavioural Ecology, 28, 760–766.

[ece38803-bib-0038] Sharon, G. , Segal, D. , Zilber‐Rosenberg, I. , & Rosenberg, E. (2011). Symbiotic bacteria are responsible for diet‐induced mating preference in *Drosophila melanogaster*, providing support for the hologenome concept of evolution. Gut Microbes, 2(3), 190–192. 10.4161/gmic.2.3.16103 21804354

[ece38803-bib-0039] Shifferman, E. M. (2012). It’s all in your head: The role of quantity estimation in sperm competition. Proceedings of the Royal Society B: Biological Sciences, 279(1730), 833–840. 10.1098/rspb.2011.2256 PMC3259941 22171084

[ece38803-bib-0040] Tan, C. K. , Doyle, P. , Bagshaw, E. , Richardson, D. S. , Wigby, S. , & Pizzari, T. (2016). The contrasting role of male relatedness in different mechanisms of sexual selection in red junglefowl. Evolution, 71(2), 403–420. 10.1111/evo.13145 PMC5324671 27925168

[ece38803-bib-0041] Tan, C. K. , Lovlie, H. , Greenway, E. , Goodwin, S. F. , Pizzari, T. , & Wigby, S. (2013). Sex‐specific responses to sexual familiarity, and the role of olfaction in Drosophila. Proceedings of the Royal Society B: Biological Sciences, 280(1771), 20131691. 10.1098/rspb.2013.1691 PMC3790479 24068355

[ece38803-bib-0042] Teseo, S. , Veerus, L. , Moreno, C. , & Mery, F. (2016). Sexual harassment induces a temporary fitness cost but does not constrain the acquisition of environmental information in fruit flies. Biology Letters, 12(1), 20150917. 10.1098/rsbl.2015.0917 26763219 PMC4785929

[ece38803-bib-0043] Wigby, S. , & Chapman, T. (2004). Female resistance to male harm evolves in response to manipulation of sexual conflict. Evolution, 58, 1028–1037. 10.1111/j.0014-3820.2004.tb00436.x 15212383

[ece38803-bib-0044] Wigby, S. , & Chapman, T. (2005). Sex peptide causes mating costs in female *Drosophila melanogaster* . Current Biology, 15(4), 316–321. 10.1016/j.cub.2005.01.051 15723791

[ece38803-bib-0045] Wild, G. , Pizzari, T. , & West, S. A. (2011). Sexual conflict in viscous populations: The effect of the timing of dispersal. Theoretical Population Biology, 80(4), 298–316. 10.1016/j.tpb.2011.09.002 21982746

[ece38803-bib-0046] Wu, C. I. , Hollocher, H. , Begun, D. J. , Aquadro, C. F. , Xu, Y. , & Wu, M. L. (1995). Sexual isolation in *Drosophila melanogaster*: A possible case of incipient speciation. Proceedings of the National Academy of Sciences of the United States of America, 92(7), 2519–2523. 10.1073/pnas.92.7.2519 7708677 PMC42249

[ece38803-bib-0054] Yun, L. , Chen, P. , Chen, J. , Singh, A. , Agrawal, A. , Aneil F, R. , & Howard, D. (2017). The physical environment mediates male harm and its effect on selection in females. Proceedings of the Royal Society B, 284, 20170424. 28679725 10.1098/rspb.2017.0424 PMC5524491

[ece38803-bib-0053] Yun, L. , Chen, P. J. , Kwok, K. E. , Angell, C. S. , Rundle, H. D. , & Agrawal, A. F. (2018). Competition for mates and the improvement of nonsexual fitness. Proceedings of the National Academy of Sciences, 115(26), 6762–6767. 10.1073/pnas.1805435115 PMC6042133 29891650

