# Supplementary material for: No evidence that relatedness or familiarity modulates male harm in Drosophila melanogaster flies from a wild population
Source: Ecol Evol. 2022 Apr 11;12(4):e8803. doi: 10.1002/ece3.8803 (PMC8995922; doi:10.1002/ece3.8803)
Supplement: Supplementary file 1 — Appendix S1 [file ECE3-12-e8803-s001.docx]

**APPENDIX**

**No evidence that relatedness or familiarity modulate male harm in *Drosophila melanogaster* flies from a wild population.**

Ana Marquez-Rosado^1†^, Clara Garcia-Co^1†^, Claudia Londoño-Nieto^1^ & Pau Carazo^1*^

1. Ethology lab, Cavanilles Institute of Biodiversity and Evolutionary Biology, University of Valencia, Valencia, Spain.

† authors contributed equally to this manuscript.

* Corresponding author: Pau Carazo, Cavanilles Institute of Biodiversity and Evolutionary Biology, c/ Catedrático José Beltrán 2, 46980, Paterna (Valencia), Spain. Telephone: +34 3544051, e-mail: pau.carazo@uv.es.

**Figure S1.** Scheme of how we set up related male pairs. We transferred 15-20 eggs from each singly-mated female into two vials containing different rearing media, at controlled density. We then combined males from the same mother that had been reared together in the same food or separately in different food to set up pairs of males that were related-familiar or related-unfamiliar.


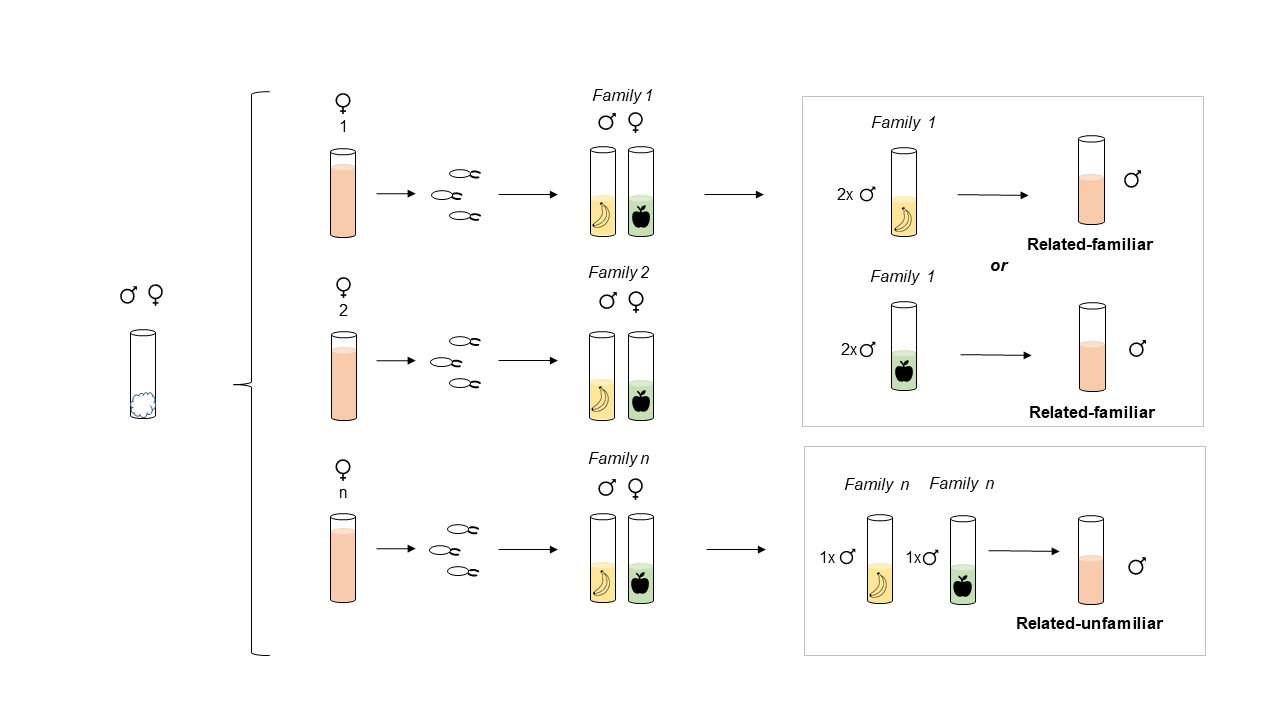


**Figure S2.** Scheme of how we set up unrelated vials. We transferred one egg from each singly-mated female into 15-20 different vials of each of the two different rearing media. By doing the same with eggs form other singly-mated females, we thus created vials with 15-20 eggs, each from a different mother. We then combined males that had been reared together in the same food (but came from different mothers), or that had been reared separately in different food (and came from different mothers) to set up pairs of males that were unrelated-familiar or unrelated-unfamiliar.


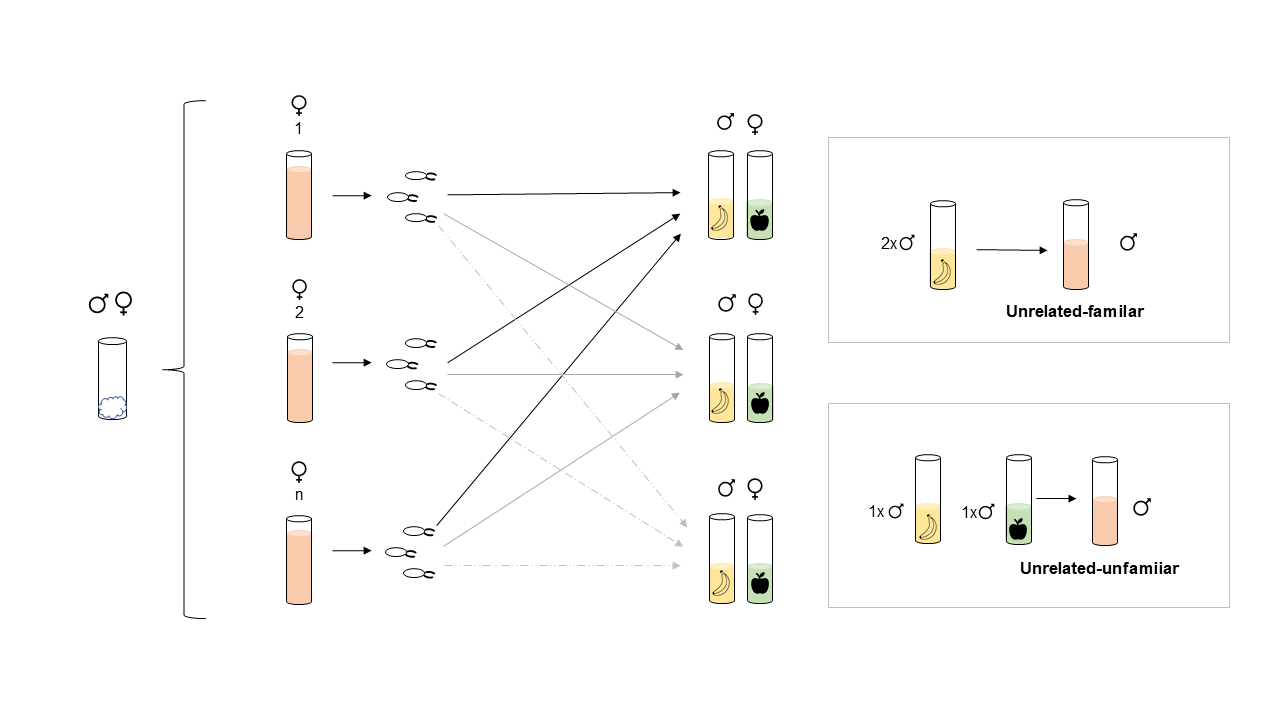


**Table S1.** Partial ethogram with behaviours measured during trials. Courtship behaviours are ordered following the most common sequence of appearance. The rest of the behaviours are sorted alphabetically. All the information used to generate this ethogram has been extracted from Sturtevant, 1915; Bastock & Manning, 1955 and Chen et al., 2002.

**Figure S3.** *In a pilot study, we left recently mated females to lay eggs in vials containing either of three experimental diets (i.e. mashed pear –P–, mashed banana –B– and mashed apple –A–) for 48h, then examined oviposition rates and egg-to-adult viability. We found no significant differences in either total eggs laid (F_2, 30_ = 0.138, p = 0.872) or adults emerged (F_2,30_ = 0.386, p = 0.280). Accordingly, we selected apple and banana as diets for our experiment.*
